# Chrono-Nutrition in Gestational Diabetes Mellitus: Implications of Meal Timing and Nutrient Distribution for Glycemic Control

**DOI:** 10.3390/nu18050712

**Published:** 2026-02-24

**Authors:** Stefania Triunfo

**Affiliations:** Department of Obstetrics and Gynecology, ASST Santi Paolo Carlo, University of Milan, 20142 Milan, Italy; stefania.triunfo@unimi.it

**Keywords:** gestational diabetes mellitus, chrono-nutrition, meal timing, circadian rhythm, glycemic control, pregnancy nutrition

## Abstract

Gestational diabetes mellitus (GDM), one of the most common metabolic complications of gestation, affects approximately 10–15% of all pregnancies and represents a significant challenge for obstetricians and diabetologists aiming to reduce adverse feto-maternal outcomes. Medical nutrition therapy remains the cornerstone of GDM management, alongside lifestyle modification and pharmacological treatment in the presence of unmet glycemic targets. However, current dietary recommendations primarily emphasize nutrient composition and caloric intake, often without fully considering the temporal aspects of food intake. Chrono-nutrition is an emerging field that investigates the interaction between meal timing, circadian rhythms, and metabolic regulation. Increasing evidence indicates that glucose metabolism and insulin sensitivity exhibit marked diurnal variations, which may be further amplified in women with GDM, resulting in time-dependent differences in postprandial glycemic responses. This narrative review summarizes the current evidence on the role of chrono-nutrition in GDM by integrating mechanistic insights with findings from observational and interventional human studies. Although the available literature is limited by heterogeneity and a paucity of well-designed randomized controlled trials, the convergence of biological plausibility and emerging clinical data suggests that chrono-nutrition may represent a potential low-risk refinement of standard medical nutrition therapy. Incorporating temporal aspects of eating into dietary counseling may help frame glycemic management within a more physiologically aligned and personalized nutritional approach for pregnancies complicated by GDM.

## 1. Introduction

Gestational diabetes mellitus (GDM) affects a substantial proportion of pregnancies worldwide and represents a major contributor to maternal and neonatal morbidity, being associated with increased risks of hypertensive disorders of pregnancy, abnormal fetal growth, cesarean delivery, and neonatal metabolic complications [1,2,3,4,5,6]. This condition is of growing interest to healthcare systems because of its association with adverse health outcomes in adulthood, particularly an elevated risk of cardiometabolic diseases [7,8]. Beyond pregnancy, women with a history of GDM exhibit a significantly increased lifetime risk of developing type 2 diabetes mellitus and cardiovascular disease, while their offspring are predisposed to obesity and metabolic dysfunction later in life, highlighting the intergenerational impact of hyperglycemia and altered glycemic exposure during gestation [7,8,9,10]. Achieving optimal glycemic control during pregnancy therefore represents a primary therapeutic objective to reduce both short- and long-term adverse outcomes [11,12,13].

In recent years, increasing attention has been directed toward temporal patterns of eating as independent determinants of metabolic health. Beyond total caloric intake and macronutrient composition, meal timing and daily nutrient distribution have emerged as critical modulators of glucose homeostasis, insulin sensitivity, and hormonal regulation. Experimental and clinical studies consistently indicate that identical meals consumed at different times of the day elicit markedly different metabolic responses, reflecting the circadian regulation of glucose metabolism [14,15,16,17]. Chrono-nutrition, an emerging field integrating principles of circadian biology into nutritional science, investigates how the timing of food intake interacts with endogenous circadian rhythms to influence metabolic regulation [17,18].

Pregnancy represents a unique metabolic condition characterized by progressive insulin resistance and dynamic adaptations in endocrine and circadian physiology. These physiological changes may increase susceptibility to circadian misalignment and amplify the metabolic consequences of mistimed nutrient intake. Growing evidence indicates that chrono-nutritional behaviors during pregnancy, such as delayed eating patterns, night-time energy intake, irregular meal timing, and altered meal frequency, may be associated with maternal metabolic dysregulation and adverse perinatal outcomes [19,20,21]. Observational studies have shown that later timing of energy intake and night-eating patterns during pregnancy are associated with impaired glucose tolerance and unfavorable metabolic profiles, suggesting that temporal eating patterns may influence glycemic regulation beyond nutrient composition alone [17]. Moreover, variability in daily eating schedules, a phenomenon often referred to as “eating jetlag,” has been associated with impaired glucose regulation and increased metabolic risk, providing a biological rationale for considering meal timing regularity as a clinically relevant factor [22].

In this context, GDM may represent a clinical model in which the interaction between circadian rhythms and nutrition becomes particularly relevant. Although emerging pregnancy-specific evidence supports a role for meal timing and daily nutrient distribution in modulating glycemic control, the temporal aspects of eating behavior remain underrepresented in both clinical research and dietary recommendations for GDM [17,20,23,24]. A clearer understanding of chrono-nutrition in pregnancy may help explain interindividual variability in glycemic control and may inform the development of more physiologically aligned and personalized nutritional strategies.

Unlike prior reviews that examined chrono-nutrition broadly during pregnancy, this review specifically focuses on GDM as a high-risk metabolic model, integrates emerging continuous glucose monitoring data, and proposes a cautious, clinically translatable conceptual framework grounded in circadian physiology.

## 2. Methods: Literature Search and Study Selection

´This narrative review was conducted to synthesize current evidence on the role of chrono-nutrition in GDM, integrating mechanistic insights from circadian biology with clinical data derived from observational and interventional human studies. A comprehensive literature search was performed using PubMed/MEDLINE and Scopus databases. Articles published in English between January 2010 and December 2025 were considered. The starting date was selected to reflect the period during which chrono-nutrition and circadian regulation of metabolism emerged as structured and clinically relevant research fields applicable to nutritional interventions. The search strategy combined terms related to GDM and chrono-nutrition, including “*gestational diabetes mellitus*,” “*pregnancy*,” “*chrono-nutrition*,” “*meal timing*,” “*circadian rhythm*,” “*carbohydrate distribution*,” “*glycemic control*,” and “*continuous glucose monitoring*.”

Eligible studies included observational studies, randomized controlled trials, systematic reviews, and relevant narrative reviews conducted in pregnant women, with a specific focus on GDM when available. Experimental animal studies, studies conducted exclusively in non-pregnant populations, conference abstracts without full text, and articles not addressing temporal aspects of nutrition were excluded. Titles and abstracts were screened by the author for relevance, and full texts were reviewed when appropriate. Given the narrative design of the review, no formal quality scoring was applied.

Given the heterogeneity of study designs, exposures, and outcome measures, a qualitative narrative synthesis was performed rather than a quantitative meta-analysis. Evidence was organized thematically, integrating biological plausibility, circadian mechanisms, and clinical relevance to provide a comprehensive and balanced overview of the current state of knowledge on chrono-nutrition in GDM.

In the context of this review, the term “chrono-nutrition” is used as an umbrella concept encompassing several related but distinct dimensions of eating behavior that interact with circadian biology. Specifically, meal timing refers to the clock time at which food intake occurs across the day, meal frequency describes the number and regularity of eating occasions, and macronutrient distribution indicates how energy and nutrients—particularly carbohydrates—are allocated across different times of day. Circadian alignment, in turn, refers to the degree to which eating patterns are synchronized with endogenous circadian rhythms and daily variations in metabolic capacity. These constructs are conceptually distinct but often interrelated, and their combined influence is considered within the chrono-nutrition framework discussed in this review.

## 3. Circadian Regulation of Glucose Metabolism in Pregnancy

**Figure 1:**
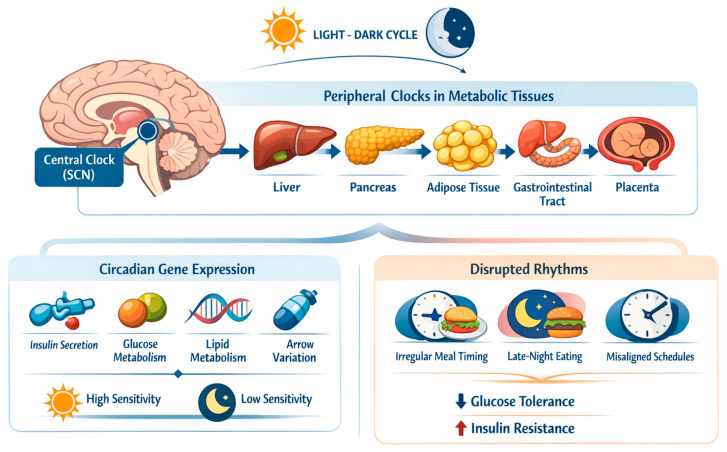
Schematic representation of circadian regulation of glucose metabolism and insulin sensitivity. The figure illustrates the interaction between the central circadian clock and peripheral metabolic clocks in regulating daily variations in glucose homeostasis. This schematic is intended to summarize established physiological mechanisms rather than depict empirically validated pathways. Created with BioRender.com.

Circadian rhythms are endogenous ~24 h cycles that regulate metabolic, hormonal, and behavioral processes and are coordinated by a central pacemaker located in the suprachiasmatic nucleus (SCN) of the hypothalamus, which synchronizes the peripheral clocks expressed in key metabolic tissues, including the liver, pancreas, adipose tissue, gastrointestinal tract, and placenta [24,25,26,27]. While the central clock is mainly entrained by the light–dark cycle [25], peripheral metabolic clocks are strongly modulated by behavioral cues [26], with feeding timing acting as one of the most potent zeitgebers [18], as depicted in Figure 1.

At the molecular level, circadian clocks regulate the rhythmic expression of genes involved in insulin secretion, insulin signaling, hepatic gluconeogenesis, glucose transport, and lipid metabolism [24,28,29]. Consequently, glucose tolerance and insulin sensitivity exhibit pronounced diurnal variation, even in healthy individuals, with reduced insulin sensitivity typically observed during the biological night [24,30,31]. Circadian misalignment-such as that induced by irregular meal timing, late eating, or misalignment between feeding schedules and endogenous rhythms has been shown to impair glucose metabolism and promote insulin resistance [18,24,30,31].

Pregnancy represents a unique physiological state characterized by meaningful endocrine and metabolic adaptations aimed at supporting fetal growth. Progressive insulin resistance develops across gestation, largely mediated by placental hormones such as human placental lactogen, progesterone, cortisol, and growth hormone [32,33,34]. These pregnancy-related changes interact with circadian regulation of metabolism, potentially amplifying vulnerability to circadian misalignment and time-dependent metabolic stress [24,27,28,30].

**Figure 2:**
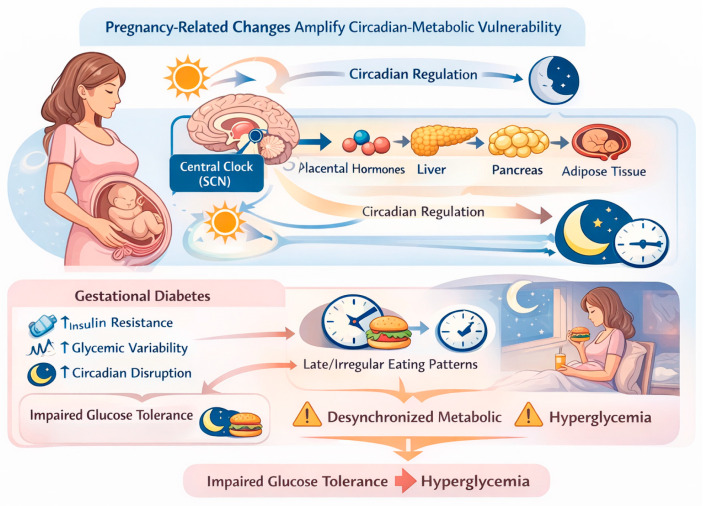
Conceptual framework illustrating circadian–metabolic vulnerability during pregnancy and GDM. The figure depicts how physiological insulin resistance of pregnancy may interact with circadian misalignment to exacerbate glycemic dysregulation in GDM. This representation is conceptual and hypothesis-based. Created with BioRender.com.

In women with GDM, circadian regulation of glucose metabolism appears further altered. Clinical evidence indicates that diurnal variation in insulin sensitivity and glucose tolerance is accentuated in GDM, resulting in marked time-of-day–dependent differences in postprandial glycemic responses [23,30,31,35]. Late or irregular eating patterns may exacerbate this dysregulation by desynchronizing peripheral metabolic clocks, leading to impaired insulin action, increased hepatic glucose output, and greater glycemic variability [24,30,36], as depicted in Figure 2.

In line with this physiological framework, a growing body of human evidence, including systematic review data, suggests that delayed meal timing, increased evening carbohydrate consumption, and reduced overnight fasting are associated with less favorable glycemic control in women with GDM [17,20,22,23]. Furthermore, chrono-nutrition–based interventions targeting meal timing and daily carbohydrate distribution have been reported to improve maternal glycemic profiles in small and heterogeneous studies, supporting the potential clinical relevance of circadian regulation as a modifiable determinant of glucose metabolism during pregnancy [35,37,38,39].

Taken together, these observations suggest that circadian regulation may represent an important, yet underrecognized, component of glucose homeostasis during pregnancy and that disruption of temporal metabolic organization may contribute to the pathophysiology and clinical expression of GDM.

## 4. Chrono-Nutrition and Glycemic Control in GDM

**Figure 3:**
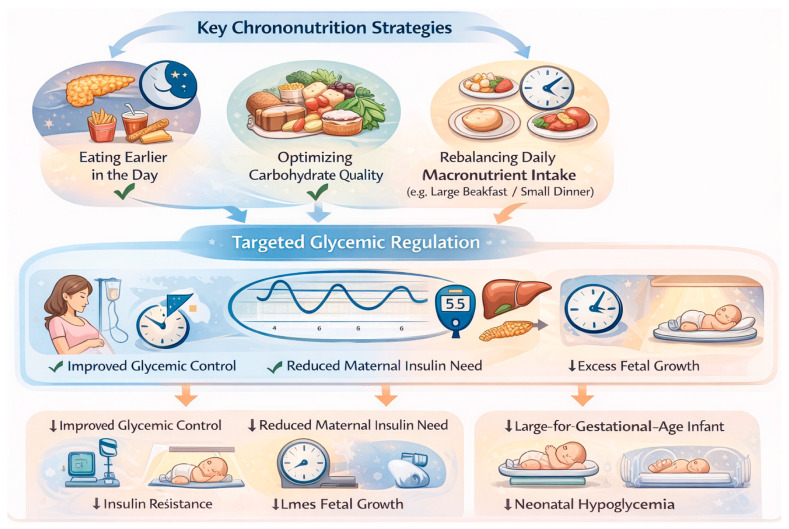
Conceptual framework linking chrono-nutrition to glycemic regulation in GDM. The figure summarizes the hypothesized relationships between meal timing, circadian alignment, and maternal glycemic patterns. It is intended to illustrate mechanistic and physiological rationale rather than provide clinical recommendations. Created with BioRender.com.

Chrono-nutrition offers a conceptual and practical framework for understanding how the timing and daily distribution of food intake interact with circadian regulation and may influence glycemic control (Figure 3).

In GDM, this interaction is particularly relevant due to the coexistence of pregnancy-induced insulin resistance and accentuated diurnal variation in glucose metabolism [16,29,30].

From a mechanistic perspective, the metabolic response to nutrient intake is strongly time dependent. The main circadian mechanisms linking meal timing to glucose metabolism and their clinical implications in GDM are summarized in Table 1.

Insulin sensitivity, pancreatic β-cell responsiveness, incretin secretion, and hepatic glucose production are regulated in a circadian manner, resulting in differential glycemic responses to identical meals consumed at different times of the day [24,29,30]. During pregnancy, these physiological rhythms are generally preserved, but may be amplified, particularly in women with GDM, leading to exaggerated postprandial hyperglycemia during periods of reduced insulin sensitivity, especially in the late afternoon and evening [23,27,30,35,36].

Emerging clinical evidence suggests that chrono-nutrition may be relevant for glycemic management in GDM. Observational studies and systematic reviews indicate that delayed breakfast timing and later initiation of daily food intake are associated with poorer glycemic control during pregnancy, independent of total energy intake and macronutrient composition [17,20].

Delayed morning feeding may prolong the overnight fasting state, exacerbate hepatic glucose output, and delay synchronization of peripheral metabolic clocks, thereby impairing postprandial glucose handling at the first meal of the day [18,26,30].

In addition to breakfast timing, evening carbohydrate intake has emerged as a critical determinant of glycemic control in GDM. Higher carbohydrate consumption during the evening, when insulin sensitivity is physiologically reduced, has been associated with increased postprandial glucose excursions and greater glycemic variability [23,30,31,35,37]. Mechanistically, late-day carbohydrate loading coincides with diminished insulin-mediated glucose disposal and altered circadian regulation of hepatic glucose production, promoting prolonged hyperglycemia and potentially increasing fetal glucose exposure [24,30,31,40].

Interventional evidence provides preliminary support for the potential role of meal timing in glycemic regulation. A randomized controlled trial combining chrono-nutrition-based dietary counseling with sleep hygiene interventions demonstrated a significant reduction in the risk of suboptimal glycemic control in women with GDM, with reduced evening carbohydrate intake identified as the primary mediator of this effect [35]. Importantly, these improvements were achieved without substantial changes in total energy intake, underscoring the importance of temporal nutrient distribution rather than caloric restriction per se.

Beyond postprandial glycemia, chrono-nutrition may also influence fasting glucose levels, which represent a common therapeutic challenge in GDM. Late-night eating or inappropriate nocturnal meal timing may disrupt the balance between hepatic glucose production and insulin-mediated suppression, contributing to elevated fasting glucose concentrations [24,30,32]. Conversely, appropriately timed evening meals or snacks with a low glycemic load may help stabilize overnight glucose metabolism, although evidence specific to GDM remains limited and warrants further investigation.

Importantly, chrono-nutrition is best conceptualized as an adjunct to standard medical nutrition therapy rather than as a standalone intervention. By aligning nutrient intake with periods of greater metabolic efficiency, chrono-nutrition-based strategies may reduce glycemic excursions, decrease glycemic variability, and potentially limit the need for pharmacological escalation in selected patients with GDM [12,35,37].

Taken together, available evidence indicates that meal timing and daily carbohydrate distribution represent modifiable and clinically meaningful determinants of glycemic control in GDM. While further well-designed randomized controlled trials are required to define optimal chrono-nutrition protocols, current data support the integration of temporal dietary guidance into nutritional counseling for women with GDM.

Key human studies investigating chrono-nutrition-related exposures and interventions in relation to glycemic regulation during pregnancy and GDM are summarized in Table 2, which provides a structured overview of study design characteristics, primary glycemic outcomes, and level of evidence. Maternal and neonatal outcomes are reported as described in the original publications, although most of them were not specifically designed or powered to assess hard obstetric or neonatal endpoints.

## 5. Meal Timing, Glycemic Variability, and Fetal Implications

Beyond mean glucose levels, increasing evidence indicates that glycemic variability and postprandial glucose excursions represent critical determinants of fetal exposure to maternal hyperglycemia and may independently contribute to adverse neonatal outcomes. Data derived from continuous glucose monitoring (CGM) studies demonstrate that larger postprandial glucose excursions and greater glycemic variability are associated with increased risks of excessive fetal growth and adverse perinatal outcomes, even when mean glycemic indices fall within recommended targets [37,40,41,42]. In GDM, exaggerated postprandial glycemic peaks are common and may occur despite apparently acceptable average glucose values, underscoring the clinical relevance of dynamic, time-dependent glucose fluctuations beyond static measures of glycemia [37,40,41,42,43].

From a physiological standpoint, maternal glucose freely crosses the placenta via facilitated diffusion, whereas insulin does not. Consequently, transient elevations in maternal glucose concentrations directly translate into increased fetal glucose exposure, stimulating fetal pancreatic β-cell hyperplasia and hyperinsulinemia. This mechanism—classically described as fuel-mediated teratogenesis—is central to the pathogenesis of excessive fetal growth, altered body composition, and long-term metabolic programming in pregnancies complicated by diabetes [10,34,40]. Importantly, fetal hyperinsulinemia appears to be driven not only by sustained maternal hyperglycemia, but also by recurrent postprandial glucose excursions, which may exert a disproportionate biological effect on the developing fetus [34,40,41].

Temporal clustering of carbohydrate intake during periods of reduced maternal insulin sensitivity—such as the late evening or biological night—may further exacerbate postprandial hyperglycemia and prolong fetal exposure to elevated glucose concentrations. Circadian variation in maternal insulin action and hepatic glucose production may amplify these effects, resulting in disproportionate glycemic excursions relative to total carbohydrate intake [24,30,40]. This interaction provides a mechanistic rationale for the potential role of chrono-nutrition in modulating fetal growth trajectories through its effects on postprandial glucose dynamics.

Although direct interventional evidence linking chrono-nutrition strategies to neonatal outcomes in GDM remains limited, available physiological and CGM-based data support the hypothesis that reducing glycemic variability and postprandial excursions—through optimized meal timing and carbohydrate distribution—could attenuate fetal hyperinsulinemia and excessive fetal growth. From this perspective, chrono-nutrition-based approaches may represent a valuable complement to traditional glycemic targets, shifting the focus from mean glucose values alone toward the temporal pattern of maternal glycemia.

## 6. Fetal Programming and Intergenerational Implications

Beyond immediate glycemic control, the potential implications of chrono-nutrition for fetal programming warrant careful consideration. Maternal glucose availability is a critical determinant of the intrauterine metabolic environment, and emerging evidence indicates that not only chronic hyperglycemia, but also recurrent postprandial glucose excursions may influence fetal insulin secretion, adiposity, and long-term metabolic trajectories [10,34,40]. In this context, glycemic variability-strongly modulated by meal timing and the diurnal distribution of carbohydrate intake-may represent an underrecognized contributor to fetal metabolic programming [34,35,37].

Chrono-nutrition-based strategies aimed at attenuating late-day hyperglycemia and aligning nutrient intake with periods of greater maternal insulin sensitivity may theoretically reduce fetal exposure to excessive glucose and insulin [30,35,40]. Such temporal modulation of maternal metabolism could influence placental nutrient transport and fetal endocrine signaling, with potential downstream effects on birth weight, body composition, and long-term cardiometabolic risk [10,28,33,40]. Although direct evidence linking chrono-nutrition interventions during pregnancy to long-term offspring outcomes remains limited, this hypothesis is biologically plausible and consistent with established models of the developmental origins of health and disease [7,10,34].

From an intergenerational perspective, improving maternal glycemic patterns through physiologically aligned nutritional strategies may confer benefits that extend beyond pregnancy. Given the well-established association between GDM and future metabolic disease in both mothers and their offspring, chrono-nutrition represents a low-risk and potentially scalable approach to modulate metabolic risk trajectories across generations [7,8,9,10]. Future longitudinal and interventional studies are required to determine whether optimization of meal timing during pregnancy can translate into sustained improvements in offspring metabolic health.

## 7. Clinical Implications and Translation into Practice

Chrono-nutrition represents a biologically plausible and potentially low-burden refinement of standard medical nutrition therapy in GDM. Rather than modifying total energy intake or macronutrient composition, this framework emphasizes the temporal alignment between nutrient intake and maternal metabolic rhythms.

Within current evidence constraints, chrono-nutrition should be considered an adjunctive and hypothesis-driven approach rather than a stand-alone or guideline-level intervention. Its potential clinical value lies in optimizing maternal glycemic patterns—particularly postprandial excursions and glycemic variability—while preserving flexibility and individualization of care.

Practical considerations that may be cautiously integrated into nutritional counseling include:

- Early initiation of daily food intake, avoiding delayed or skipped breakfast;
- Redistribution of carbohydrate intake toward earlier daytime hours, when insulin sensitivity is physiologically higher;
- Avoidance of late-night eating, which may exacerbate nocturnal insulin resistance;
- Maintenance of regular meal timing across days to reduce circadian misalignment;
- Individualized management of evening snacks, favoring low glycemic load options when required.

**Figure 4:**
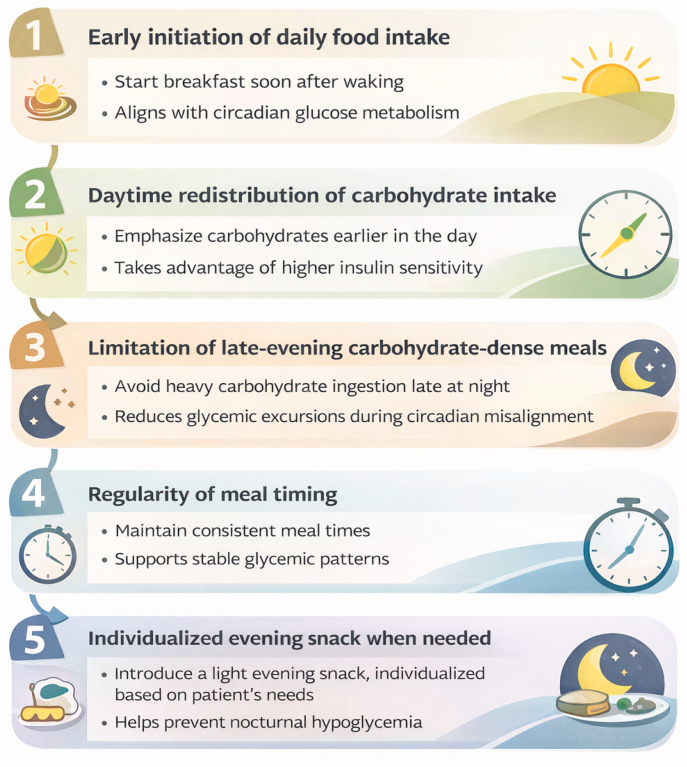
Conceptual framework summarizing practical chrono-nutrition considerations in GDM. These principles are hypothesis-driven and based on current mechanistic knowledge and emerging human evidence. They are intended to translate the conceptual framework into clinical practice by providing non-prescriptive, flexible considerations that complement standard medical nutrition therapy and should be individualized according to clinical context. Created with BioRender.com.

These principles are schematically summarized in Figure 4 and are intended as flexible, non-prescriptive considerations that complement standard medical nutrition therapy. Adaptation to gestational age, severity of hyperglycemia, pharmacological treatment, sleep–wake patterns, and cultural eating behaviors remains essential.

## 8. Limitations and Future Research

The current evidence base on chrono-nutrition in GDM remains methodologically limited. Most available data derive from observational studies and small, heterogeneous interventional trials, precluding robust causal inference. Study designs, populations, and outcome measures vary substantially, and standardized definitions of meal timing and glycemic variability are lacking. Assessment of chrono-nutrition-related behaviors frequently relies on self-reported dietary data, increasing susceptibility to recall bias.

Only a subset of studies reports obstetric or neonatal outcomes (Table 2), which are inconsistently assessed and rarely powered as primary endpoints [17,35,44]. Direct interventional evidence demonstrating improvement in hard maternal or neonatal outcomes (e.g., cesarean delivery, hypertensive disorders, preterm birth, neonatal hypoglycemia, neonatal intensive care admission) remains lacking [12,17,44]. Long-term offspring metabolic consequences have not been systematically evaluated, and potential intergenerational implications remain speculative.

Most studies have been conducted in specific populations and healthcare settings, limiting generalizability. In addition, as a narrative review, the present synthesis does not follow a systematic or meta-analytic methodology and may be subject to selection bias.

Future research should prioritize adequately powered randomized controlled trials integrating chrono-nutrition strategies into standard medical nutrition therapy, incorporating continuous glucose monitoring and standardized definitions of glycemic variability. Longitudinal studies with extended maternal and offspring follow-up are required to clarify whether temporally aligned dietary strategies can meaningfully influence fetal growth patterns and long-term metabolic risk.

## 9. Conclusions

Chrono-nutrition provides a biologically coherent and physiologically grounded framework for integrating temporal eating patterns into the nutritional management of GDM. Emerging human evidence suggests that meal timing and daily carbohydrate distribution may influence maternal glycemic dynamics—particularly postprandial excursions and glycemic variability—beyond total caloric intake and macronutrient composition alone.

However, current data remain predominantly observational and interventional evidence is limited to small, heterogeneous trials. Causal effects on obstetric and neonatal outcomes have not yet been established. Within these constraints, chrono-nutrition should be regarded as an adjunctive, low-risk, and hypothesis-driven refinement of standard medical nutrition therapy rather than a guideline-level intervention.

Future research priorities include adequately powered randomized controlled trials integrating continuous glucose monitoring, standardized definitions of meal timing constructs, and comprehensive assessment of maternal and neonatal endpoints. Longitudinal follow-up studies are required to determine whether temporally aligned nutritional strategies during pregnancy translate into sustained metabolic benefits for mothers and offspring. Until such data are available, chrono-nutrition represents a promising but still evolving paradigm aimed at aligning maternal metabolism with circadian physiology in pregnancies complicated by GDM.

## Figures and Tables

**Table 1:** Circadian mechanisms linking meal timing to glycemic regulation and clinical implications in GDM.

Circadian Mechanism	Time-of-Day Variation	Effect on Glucose Metabolism	Clinical Relevance in GDM
Insulin Sensitivity	Higher in morning, lower in evening/night	Reduced glucose disposal during late-day meals	Evening carbohydrate intake associated with higher postprandial glucose excursions
β-cell responsiveness	Peaks earlier in the day	Blunted insulin secretion later in the day	Exaggerated postprandial hyperglycemia in afternoon/evening
Hepatic glucose production	Increased during biological night	Higher fasting and preprandial glucose levels	Late eating may worsen fasting hyperglycemia
Incretin Secretion	Diurnal variation favoring daytime	Reduced incretin effect in evening	Impaired postprandial glucose control with late meals
Peripheral clock synchronization	Entrained by feeding timing	Irregular or late meals desynchronize metabolic clocks	Increased glycemic variability and insulin resistance

**Table 2:** Chrono-nutrition in pregnancy and GDM: structured synthesis of human evidence on glycemic dynamics, and maternal–neonatal outcomes.

Author (Year)	Country	Study Design	Population (*n*)	Intervention/ Exposure	Timing of Intervention	Primary Glycemic Outcome	CGM Used	Main Findings	Maternal/ Neonatal Outcomes Reported
Wang et al. (2019) [20]	USA	Prospective cohort	Pregnant women (≈1000)	Observational: timing of daily energy intake	Mid- pregnancy	OGTT	No	Highest tertile of late-day energy intake associated with increased risk of glucose intolerance (adjusted OR ≈ 1.5–1.7)	Not assessed
Gontijo et al. (2020) [21]	Brasil	Observational Cohort	Pregnant women (100)	Observational: timing of food intake	I–II–III Trimester	Not primary glycemic outcome (diet quality, GWG)	No	Later eating timing associated with lower diet quality scores and greater GWG (≈+1.2 kg)	GWG
Messika et al. (2022) [23]	Israel	Randomized controlled trial	Women with GDM (90)	Chrono-nutrition counseling + sleep hygiene	From GDM diagnosis	Mean CGM glucose; TAR; glycemic variability	Yes	Lower mean CGM glucose and reduced time above range; evening carbohydrate intake reduced by ≈20%	Heterogeneous and inconsistently reported perinatal outcomes
Messika et al. (2024) [35]	Israel	Observational study	Women with GDM (246)	Observational: meal timing and chronobiological factors	From GDM diagnosis to delivery	CGM (TAR, SD, MAGE)	Yes	Later eating timing associated with higher mean glucose and increased glycemic variability (SD, MAGE)	Not powered for obstetric or neonatal outcomes
Boege et al. (2025) [17]	Multiple	Systematic review	Pregnant women (16 studies)	Observational and interventional studies	Variable across pregnancy	Postprandial glucose; glycemic variability (heterogeneous measures)	Variable across studies	Later meal timing associated with poorer glycemic outcomes in 11 of 16 studies, mainly affecting postprandial glucose and glycemic variability; no pooled effect estimates available	Exploratory analyses of birth weight and neonatal anthropometrics

CGM, continuous glucose monitoring; OGTT, oral glucose tolerance test; GWG, gestational weight gain; LGA, large for gestational age; TAR, Time Above Range; SD, standard deviation; MAGE, mean amplitude of glycemic excursions.

## Data Availability

No new data were created or analyzed in this study.

## Abbreviations

GDM	Gestational diabetes mellitus
MNT	Medical nutrition therapy
CGM	Continuous glucose monitor
MAGE	Mean amplitude of glycemic excursions.

## References

[1] American Diabetes Association (2024). Management of diabetes in pregnancy: Standards of Care in Diabetes—2024. Diabetes Care.

[2] McIntyre H.D., Catalano P., Zhang C., Desoye G., Mathiesen E.R., Damm P. (2019). Gestational diabetes mellitus. Nat. Rev. Dis. Primers.

[3] Landon M.B., Spong C.Y., Thom E., Carpenter M.W., Ramin S.M., Casey B., Wapner R.J., Varner M.W., Rouse D.J., Thorp J.M. (2009). A multicenter, randomized trial of treatment for mild gestational diabetes. N. Engl. J. Med..

[4] Triunfo S., Lanzone A. (2014). Impact of overweight and obesity on obstetric outcomes. J. Endocrinol. Investig..

[5] Triunfo S., Lanzone A. (2015). Potential impact of maternal vitamin D status on obstetric well-being. J. Endocrinol. Investig..

[6] Triunfo S., Lanzone A., Lindqvist P.G. (2017). Low maternal circulating levels of vitamin D as potential determinant in the development of gestational diabetes mellitus. J. Endocrinol. Investig..

[7] Chen A., Tan B., Du R., Chong Y.S., Zhang C., Koh A.S., Li L.-J. (2024). Gestational diabetes mellitus and development of intergenerational overall and subtypes of cardiovascular diseases: A systematic review and meta-analysis. Cardiovasc. Diabetol..

[8] Kramer C.K., Campbell S., Retnakaran R. (2019). Gestational diabetes and the risk of cardiovascular disease in women: A systematic review and meta-analysis. Diabetologia.

[9] Bellamy L., Casas J.-P., Hingorani A.D., Williams D. (2009). Type 2 diabetes mellitus after gestational diabetes: A systematic review and meta-analysis. Lancet.

[10] Dabelea D., Crume T. (2011). Maternal Environment and the Transgenerational Cycle of Obesity and Diabetes. Diabetes.

[11] Metzger B.E., Gabbe S.G., Persson B., Buchanan T.A., Catalano P.A., Damm P., Dyer A.R., Leiva Ad Hod M., Kitzmiler J.L., International Association of Diabetes and Pregnancy Study Groups (IADPSG) (2010). Recommendations on the diagnosis and classi-fication of hyperglycemia in pregnancy. Diabetes Care.

[12] American Diabetes Association Professional Practice Committee (2025). 15. Management of Diabetes in Pregnancy: Standards of Care in Diabetes—2025. Diabetes Care.

[13] WHO (2025). WHO Recommendations on Care for Women with Diabetes During Pregnancy.

[14] Garaulet M., Gómez-Abellán P. (2013). Chronobiology and obesity. Nutr. Hosp..

[15] Johnston J.D. (2014). Physiological responses to food intake throughout the day. Nutr. Res. Rev..

[16] Reutrakul S., Van Cauter E. (2014). Interplay between sleep, circadian rhythms, and glucose metabolism. Endocr. Rev..

[17] Boege H.L., Park C., Gagnier R., Deierlein A.L. (2025). Timing of eating and glycemic control during pregnancy: A systematic review. Nutr. Metab. Cardiovasc. Dis..

[18] Flanagan A., Bechtold D.A., Pot G.K., Johnston J.D. (2020). Feeding rhythms and the circadian regulation of metabolism. Front. Nutr..

[19] Chen Y.-E., Loy S.L., Chen L.-W. (2023). Chrononutrition during Pregnancy and Its Association with Maternal and Offspring Outcomes: A Systematic Review and Meta-Analysis of Ramadan and Non-Ramadan Studies. Nutrients.

[20] Wang J.B., Patterson R.E., Ang A., Emond J.A., Shetty N., Arab L. (2019). Timing of energy intake during the day is associated with the risk of gestational glucose intolerance. BMJ Open.

[21] Gontijo C.A., Balieiro L.C.T., Teixeira G.P., Fahmy W.M., Crispim C.A., Maia Y.C.P. (2020). Effects of timing of food intake on eating patterns, diet quality and weight gain during pregnancy. Br. J. Nutr..

[22] Zerón-Rugerio M.F., Hernáez Á., Porras-Loaiza A.P., Cambras T., Izquierdo-Pulido M. (2019). Eating Jet Lag: A Marker of the Variability in Meal Timing and Its Association with Body Mass Index. Nutrients.

[23] Messika A., Toledano Y., Hadar E., Shmuel E., Tauman R., Shamir R., Froy O. (2022). Relationship among chrononutrition, sleep, and glycemic control in women with gestational diabetes mellitus: A randomized controlled trial. Am. J. Obstet. Gynecol. MFM.

[24] Panda S. (2016). Circadian physiology of metabolism. Science.

[25] Hastings M.H., Maywood E.S., Brancaccio M. (2018). Generation of circadian rhythms in the suprachiasmatic nucleus. Nat. Rev. Neurosci..

[26] Mohawk J.A., Green C.B., Takahashi J.S. (2012). Central and peripheral circadian clocks in mammals. Annu. Rev. Neurosci..

[27] Bates K., Herzog E.D. (2020). Maternal-Fetal Circadian Communication During Pregnancy. Front. Endocrinol..

[28] Waddell B.J., Wharfe M.D., Crew R.C., Mark P.J. (2012). Circadian variation in placental and metabolic function. Placenta.

[29] Saini C., Petrenko V., Pulimeno P., Giovannoni L., Berney T., Hebrok M., Howald C., Dermitzakis E.T., Dibner C. (2016). A functional circadian clock is required for proper insulin secretion by human pancreatic islet cells. Diabetes Obes. Metab..

[30] Leproult R., Holmbäck U., Van Cauter E. (2014). Circadian Misalignment Augments Markers of Insulin Resistance and Inflammation, Independently of Sleep Loss. Diabetes.

[31] Morris C.J., Purvis T.E., Mistretta J., Scheer F.A. (2016). Effects of the Internal Circadian System and Circadian Misalignment on Glucose Tolerance in Chronic Shift Workers. J. Clin. Endocrinol. Metab..

[32] Barbour L.A., McCurdy C.E., Hernandez T.L., Kirwan J.P., Catalano P.M., Friedman J.E. (2007). Cellular mechanisms for insulin resistance in normal pregnancy and gestational diabetes. Diabetes Care.

[33] Stern C., Schwarz S., Moser G., Cvitic S., Jantscher-Krenn E., Gauster M., Hiden U. (2021). Placental Endocrine Activity: Adaptation and Disruption of Maternal Glucose Metabolism in Pregnancy and the Influence of Fetal Sex. Int. J. Mol. Sci..

[34] Freinkel N. (1980). Banting Lecture 1980: Of Pregnancy and Progeny. Diabetes.

[35] Messika A., Toledano Y., Hadar E., Tauman R., Froy O., Shamir R. (2024). Chronobiological Factors Influencing Glycemic Control and Birth Outcomes in Gestational Diabetes Mellitus. Nutrients.

[36] Reiter R.J., Tan D.X., Korkmaz A., Rosales-Corral S.A. (2014). Melatonin and stable circadian rhythms optimize maternal, placental and fetal physiology. Hum. Reprod. Update.

[37] Monnier L., Colette C. (2008). Glycemic variability: Should we and can we prevent it?. Diabetes Care.

[38] Yamamoto J.M., Kellett J.E., Balsells M., García-Patterson A., Hadar E., Solà I., Gich I., van der Beek E.M., Castañeda-Gutiérrez E., Heinonen S. (2018). Gestational Diabetes Mellitus and Diet: A Systematic Review and Meta-analysis of Randomized Controlled Trials Examining the Impact of Modified Dietary Interventions on Maternal Glucose Control and Neonatal Birth Weight. Diabetes Care.

[39] Johnston J.D., Ordovás J.M., Scheer F.A., Turek F.W. (2016). Circadian Rhythms, Metabolism, and Chrononutrition in Rodents and Humans. Adv. Nutr..

[40] Desoye G., Nolan C.J. (2016). The fetal glucose steal: An underappreciated phenomenon in diabetic pregnancy. Diabetologia.

[41] Feig D.S., E Donovan L., Corcoy R., E Murphy K., A Amiel S., Hunt K.F., Asztalos E., Barrett J.F.R., Sanchez J.J., de Leiva A. (2017). Continuous glucose monitoring in pregnant women with type 1 diabetes (CONCEPTT): A multicentre international randomised controlled trial. Lancet.

[42] Resi V., Bianchi C., Burlina S., Grancini V., Manicardi E., Masulli M., Scarpitta A.M., Sorice G.P., Fresa R. (2025). The use of technology in diabetes in pregnancy: A position statement of expert opinion from the association of medical diabetologists (AMD), the Italian society of diabetology (SID) and the interassociative diabetes and pregnancy study group. Acta Diabetol..

[43] Hernández T.L., Barbour L.A. (2013). A standard approach to gestational diabetes mellitus using continuous glucose monitoring. Curr. Diab. Rep..

[44] Xega V., Liu J.L. (2025). Chrono-nutrition in gestational diabetes: Toward precision timing in maternal care. J. Pers. Med..

